# From COVID-19 Pandemic to Patient Safety: A New “Spring” for Telemedicine or a Boomerang Effect?

**DOI:** 10.3389/fmed.2022.901788

**Published:** 2022-06-15

**Authors:** Francesco De Micco, Vittorio Fineschi, Giuseppe Banfi, Paola Frati, Antonio Oliva, Guido Vittorio Travaini, Mario Picozzi, Giuseppe Curcio, Leandro Pecchia, Tommasangelo Petitti, Rossana Alloni, Enrico Rosati, Anna De Benedictis, Vittoradolfo Tambone

**Affiliations:** ^1^Bioethics and Humanities Research Unit, Campus Bio-Medico University of Rome, Rome, Italy; ^2^Department of Anatomical, Histological, Forensic and Orthopaedic Sciences (SAIMLAL), Sapienza University of Rome, Rome, Italy; ^3^IRCCS Istituto Ortopedico Galeazzi, Milan, Italy; ^4^Vita-Salute San Raffaele University, Milan, Italy; ^5^Department of Health Surveillance and Bioethics, Section of Legal Medicine, Fondazione Policlinico A. Gemelli IRCCS, Università Cattolica del Sacro Cuore, Rome, Italy; ^6^Department of Biotechnology and Science of Life, Center for Clinical Ethics, Insubria University, Varese, Italy; ^7^Department of Biotechnological and Applied Clinical Sciences, University of L'Aquila, L'Aquila, Italy; ^8^Campus Bio-Medico University of Rome, Rome, Italy; ^9^Hygiene, Public Health and Statistics, Campus Bio-Medico University of Rome, Rome, Italy; ^10^Department of Medical Affairs, Fondazione Don Carlo Gnocchi Onlus, Rome, Italy; ^11^Casa di Cura “Auxologico Roma–Buon Pastore”, Rome, Italy; ^12^Nursing Science Research Unit, Campus Bio-Medico University of Rome, Rome, Italy

**Keywords:** telemedicine, Healthcare risk Management, patient safety, quality of care (QoC), systemic clinical risk management, clinical governance (CG), Ethics of Job Well Done

## Abstract

During the Covid-19 health emergency, telemedicine was an essential asset through which health systems strengthened their response during the critical phase of the pandemic. According to the post-pandemic economic reform plans of many countries, telemedicine will not be limited to a tool for responding to an emergency condition but it will become a structural resource that will contribute to the reorganization of Healthcare Systems and enable the transfer of part of health care from the hospital to the home-based care. However, scientific evidences have shown that health care delivered through telemedicine can be burdened by numerous ethical and legal issues. Although there is an emerging discussion on patient safety issues related to the use of telemedicine, there is a lack of reseraches specifically designed to investigate patient safety. On the contrary, it would be necessary to determine standards and specific application rules in order to ensure safety. This paper examines the telemedicine-risk profiles and proposes a position statement for clinical risk management to support continuous improvement in the safety of health care delivered through telemedicine.

## Introduction

Telemedicine enables medical care in situations where distance is a critical factor by using information and communication technologies (ICT) to exchange information for the diagnosis, treatment and prevention of disease and trauma, for research and evaluation and for the continuing education of health professionals in the interests of individual and community health (1).

Compared with traditional health care, telemedicine may represent (a) a diagnostic and/or therapeutic alternative, (b) a supportive health care activity that increases efficiency and distributive equity, (c) an integrative health care intervention, (d) a health care activity able to completely replace the usual health care intervention (2).

Prior to the pandemic, telemedicine was adopted by Health Systems in various regions/countries, although in different and uneven ways (3) and it was supported by legislation and policy documents (4–7).

However, the Covid-19 health emergency greatly increased the use of telemedicine both to provide health care to Covid-19 patients with mild symptoms and to ensure that diagnostic and therapeutic health care activities were carried out while respecting the physical distance between people (8–10). For this reason, the World Health Organization (WHO) and the Organization for Economic Co-operation and Development (OECD) considered telemedicine an essential asset through which health systems strengthened their response during the critical phase of pandemic management (11, 12). In the near future, telemedicine will not only be a tool for responding to an emergency situation. Telemedicine will become a structural tool for Healthcare Systems to provide diagnostic and therapeutic services, also thanks to integration with robotics and artificial intelligence (13).

The ambitious EU4Health 2021–2027 investment programme promoted activities to enhance telemedicine and supported optimal use of telemedicine (14). Telemedicine is a cornerstone for strengthening health care and improving standards of treatment for citizens in the reform and investment plans presented by Italy, Germany and France to access Next Generation EU funds (15–17).

In the United States, the Telehealth Extension and Evaluation Act will establish an extension of telemedicine services by ensuring a thorough evaluation of these services prior to future permanent action (18).

Telemedicine can therefore contribute to a reorganization of Healthcare Services, allowing the shift of health care from the hospital to the home-based care, through innovative citizen-centered care models and facilitating access to Health Services. Therefore, telemedicine is a great resource that makes possible new approaches to care and new ways of continuity of care between hospital and home-based care (19, 20).

However, the spread of telemedicine presents Health Systems around the world with new challenges, one of the most important being patient safety. The use of digital technologies can expand risk factors.

Healthcare Risk Management is defined by the clinical and administrative activities performed to identify, assess and reduce the risk of injury to patients, staff and visitors and the risk of loss to the organization itself (21).

The aim of Clinical Risk Management (CRM) is to improve the quality and safety of health care activities by identifying and preventing conditions that could put a patient at risk of an adverse event (22).

Concerning telemedicine: have clinical risk control models been established with the aim of preventing the occurrence of an adverse event or error and limiting its consequences? Have training programmes for health workers, patients, formal caregivers and family members on risk management been set up? Has an incident reporting system been established? Have systems been established to measure risks, adverse events and all factors affecting risk?

It was pointed out that telemedicine is burdened by numerous ethical and legal issues, and that standards and specific guidelines for its application should be drawn up (23).

A literature review conducted to identify patient safety risks associated with the use of telemedicine showed that although there is an emerging discussion of patient safety issues related to the use of telemedicine, there is a lack of researches specifically designed to investigate patient safety (24).

However, evidence suggests that attention to patient safety should be an important feature to ensure integrity in the design, implementation and operation of telemedicine services (25). This topic is eminent while “digital therapies” and “digital trials” are now proposed and accepted, even by regulatory agencies.

Existing global documents frame telemedicine as part of the process of computerization and digitalization of the health system, but they do not provide a comprehensive and up-to-date framework for the new needs that have emerged. However, the definition of evidence-based eHealth standards and rules is required to ensure safety (26).

In a changing healthcare scenario characterized by the expansion of healthcare technology, hospitals are developing proactive Clinical Risk Management plans based on a much broader perspective of the entire healthcare ecosystem (27). In fact, according to the American Society for Healthcare Risk Management (ASHRM), Clinical Risk Management encompasses eight risk domains: operational, clinical and patient safety, strategic, financial, human capital, legal and regulatory, technological, environmental and infrastructure (28).

An efficient clinical risk management ensures effective planning, high standards of performance, efficient and effective resource allocation, improved competitive capacity and organizational innovation.

Like any free and responsible human act, health risk management has an intrinsic ethical value (29). Therefore, clinical risk management activities should protect healthcare organizations by fulfilling their mission, i.e., promoting and protecting the health of patients (30). The health risk management professional is responsible for helping to promote the overall quality of life, dignity, safety, and wellbeing of every individual in need of health services (31).

After analyzing the risk profiles related to the use of telemedicine, this paper aims to propose an ethical-based position paper for clinical risk management to support continuous improvement of safety in telemedicine.

## Telemedicine Risk-Related

In healthcare institutions, recognition of the full spectrum of activities performed by clinical and non-clinical staff has allowed the development of standards to improve activities and reduce the risks associated with process variability (21).

**Figure 1:**
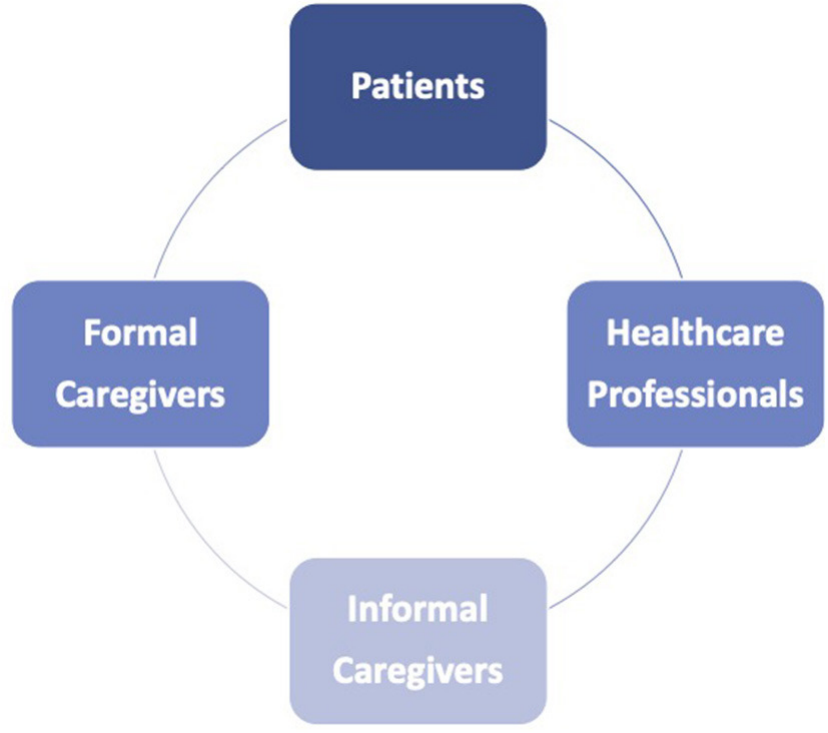
Relationships between patients, professional and non-professional stakeholders in telemedicine health care provision.

Risk assessment in telemedicine requires consideration of the professional and non-professional stakeholders involved in the process of care, as well as the particular setting in which the health care takes place. Patient safety in home-based care is related to the variables of the relationships between patients, healthcare workers (HWs), informal and formal caregivers (24, 32) (Figure 1).

## Human Factors

“To err is human” (33). Considering the operational framework of medical care in telemedicine it is evident that, as in hospital care, a first source of potential risk for patient safety is the human factor. Such is the relevance of the human factor that Human Factors and Ergonomics (HFE) is recognized by the leadership of health care institutions as a scientific discipline capable of producing knowledge to redesign Healthcare Systems and processes and improve patient safety and quality of care (34).

In telemedicine, therefore, some adverse events are entirely comparable to those that occur in any health care organization, such as deaths or injuries resulting from incorrect drug therapy or deaths or injuries due to the patient falling down. Compared to hospital care there is a significant difference. Unlike health care personnel, informal caregivers are not bound by standards and can make non-evidence-based decisions (35). However, “the conscientious, explicit and judicious use of the best current evidence in making decisions about the care of individual patients” is the main paradigm on which health care is based (36) and improving evidence-based practice is also one of the main goals for improving patient safety (37).

Therefore, the systematic inclusion in the health care process of caregivers who are not constrained by guidelines or standards of good clinical practice may significantly increase the risk level of telemedicine. Caregivers often cannot give accurate information because of miscommunication, misunderstandings or poor memory (38) and have been identified as contributors to several adverse events (39). Canadian retrospective study shows that caregivers of home care patients contribute to 20.4% of adverse events (40).

Fortunately, telemedicine itself could mitigate this “health care bias.” An intelligent telecare system could monitor whether caregivers are on time for scheduled visits, monitor their response time, whether they respond to a traditional alarm call, how long they stay for each visit, the total number of visits and the type of care provided at each visit (32). Monitoring the type and timeliness of care provided through telemedicine has a limitation because it is a quantitative assessment and cannot offer any information on “the application of medical science and technology in a manner that maximizes its benefit to health without correspondingly increasing the risk” (41). A “telemedicine-tailored” organization, a modified staff management and the identification of specific competences and responsibilities may contribute to a further attenuation of caregiver-related risks. Patient safety in telemedicine could be implemented by research and application of health care work system models such as SEIPS (34, 42), the patient-centered medical home model (43) or the work system of the patient-centered medical home (44).

Assessing the human factors issues in telemedicine is a challenge that must be taken up decisively in order to develop telemedicine-specific risk management strategies to both prevent avoidable errors and contain their possible harmful effects.

## Patient-Physician Relationships

A second potential patient safety risk concerns the physician-patient relationship.

The impact of verbal and non-verbal communication in building an empathic relationship in the processes of diagnosis, treatment and rehabilitation has been proven (45–47). Above all, communication errors are the cause of the vast majority of unexpected adverse events in patients (48).

For this reason, improving the effectiveness of communication is one of the International Patient Safety Goals (IPSG) developed by Joint Commission International (JCI) (49). Communication plays a key-role in the etiology, exacerbation and reduction of adverse events in health care.

However, digital technologies in health care could depersonalize and negatively influence the healthcare relationship (45, 50, 51), leading to poor communication and limited data transmission which can expose the patient to a clinical risk in many situations (52).

It has been shown that during a telemedicine health care service compared to an in-person health care provision, patients are more likely to ask for repeat information (53), receive less information and the specialist physician interacts more with the primary care provider (54).

These studies show some important critical issues in the physician-patient relationship during a telemedicine health care provision. However, a potential cause of risk to patient safety is that we do not currently fully understand the nature and content of the communication process (55).

Even if the matter is largely unexplored (56), a clinical risk assessment and management in telemedicine cannot disregard the high number of adverse events, or near misses, which occur due to poor or insufficient communication and which may concern both communication between HWs (internal communication) and communication between HWs and the patient or his/her caregivers (external communication) (57).

On the contrary, no matter how a health care provision is made, effective, timely, targeted, comprehensive, unambiguous and easily understood communication is mandated to reduce errors and improve patient safety.

In this regard, the World Medical Association (WMA) has recommended that telemedicine should be limited to situations where a physician cannot be physically present within a safe and acceptable period of time, or it should be used in the management of chronic conditions or in follow-up after initial treatment, if its safety and effectiveness have been demonstrated (58).

Therefore, in a priority area for the application of telemedicine models for promoting continuity of care such as the follow-up of chronic diseases, adequate physician-patient interaction is required, giving consideration to how information can be stored and accessed for future episodes of treatment in line with patients' preferences (or the decisions of their relatives or caregivers) and how the information will be transmitted to the patient's general practitioner or other physicians caring for the patient (59). It is therefore essential to identify the Primary Care Physician (PCP) as the physician responsible for the treatment and coordination of the patient with the remote medical team (58). The role of healthcare professional in the web-based interrelationship, besides a specific training and certification of knowledge, should be properly defined to assure the correct and appropriate connection, considering also the present possible modifications of professional involvement, generally named “task shifting,” owing to even decrease of medical doctors, especially in some Countries and in same specialties (60).

Given that communication between doctors and patients is fundamental for patient safety, continuous, effective and high-quality communication must be guaranteed in telemedicine (61) considering not only the doctor-patient interaction but also the participation in the care process of formal and informal caregivers.

## Informed Consent

Related to the different configuration of the physician-patient relationship and to the critical communication and information issues, a third source of potential risk to patient safety concerns the lack of protection of the patient's right to autonomy, which is expressed mainly through informed consent (62). Informed consent is an essential feature of patient-centered health care and remains central to patient safety (63).

Information and consent in telemedicine, in addition to guaranteeing the rights and duties provided for any health care treatment, should also consider the specific risks of providing healthcare using ICTs (64).

Indeed, if it is fair to say that consent to health care treatment is particularly crucial for “high risk” procedures, it is also fair to say that the use of ICTs or the “distance factor” may push routine health care treatments into a higher risk category. An incorrect or delayed diagnosis due to errors in the transmission of health documentation (medical records, X-rays and medical device printouts) or an inaccurate assessment of the patient's condition due to an incomplete physical examination because of the “distance factor” are just a few examples of adverse events in telemedicine (65).

In addition, the involvement of relatives and caregivers may be necessary in the management of disabling or chronic illnesses. This is a far from remote event, given that public health policies have generally identified telemedicine as a target for development because of its potential to treat and manage patients with chronic diseases at their homes rather than in hospital.

It is therefore necessary for the physician to customize the informed consent procedure to provide patients and their caregivers with the necessary information on the distinctive features of telemedicine (58, 59).

The information should include how the confidentiality of personal data is protected, how patient personal data are documented and stored, how medications are prescribed, how to interact with other medical specialists, procedures for activating an emergency plan, conditions under which the telemedicine health care may be interrupted and the patient referred to in-person health care, potential technical failures, etc. (66). At the same time, patients and their caregivers should be aware of the potential, limitations and modalities of telemedicine and what is expected of patients when using these technologies (58, 59).

Nevertheless, it should be considered that the patient's decision-making autonomy might be compromised when choice is limited by access or pressures from family and community (51) and also the limitations of electronic informed consent especially for that target population with insufficient IT background or low trust toward health technologies (67).

Lack of or insufficient informed consent is an important source of medical malpractice cases (68). In the near future, an increasingly large population will be treated through telemedicine, so Clinical Risk Managers in healthcare organizations should seriously consider the critical issues related to inadequate information and uninformed consent from the perspective of both patient safety and medical malpractice.

## Patient Identification

A fourth potential risk to patient safety concerns patient misidentification.

Patient identification is a crucial step in ensuring the safety of treatment and healthcare, both because it is necessary to reliably identify the person receiving the healthcare service and to verify that the healthcare provided corresponds to that individual patient (49).

Currently, most telemedicine does not allow for strong and compliant verification of patient identity (69) and it is also possible for identity theft to occur (70).

Thus, critical issues in patient identification may have important implications not only on patient safety but may also offer new fraud activities with negative economic and trust repercussions for National health systems.

In addition, patient misidentification may have as a direct consequence the transmission of sensitive data to third parties and the violation of privacy. Protection of privacy, access to data, interoperability and quality of recorded data have ethical, legal and social implications in telemedicine with major implications (45, 50, 51).

For this reason, it is necessary to ensure that personal data obtained during a telemedicine consultation must be protected by appropriate security measures and the electronic transmission of information must be safeguarded against unauthorized access (58).

However, there is a lack of standardization regarding security in telemedicine, and much research does not consider the possibility of having to diversify data security systems according to the type of population targeted by the telemedicine service. For example, a data integrity security system may be efficient in an elderly population but may fail in cognitively impaired adults (71).

One thing is certain, trust in telemedicine could be undermined if the risks to patient privacy and safety are not seriously addressed (72). Such concerns are much more urgent considering a significant growth in cybercrime attacks, during the pandemic time for Covid 19, especially against healthcare facilities (73).

## Structural and Technological Factors

In healthcare, to define the level of risk, it is also necessary to consider the safety and logistics of environments, the operation, maintenance and renewal of equipment and instruments and the critical issues of infrastructure, networks, digitalization and automation. In this scenario, particular attention should also be paid to the assessment, introduction and use of equipment and technology on the patient by non-specifically trained staff (74).

The development and implementation of ICT are creating new and different ways of doing medicine (58). Regarding patient safety, already at the beginning of the 21st century, the Institute of Medicine (IOM) argued that the use of medical technology by non-HWs would become increasingly important as healthcare shifted to the outpatient and home setting (33).

Therefore, we can consider structural and technological factors as the fifth potential risk to patient safety.

The development process and performance of these devices are influenced by an infinite number of variables that are not always considered and whose effects are not always predicted (75).

A review of the risks to patient safety in telemedicine showed that the main critical issues relate to the poor technical quality of the systems, poor usability of the technology, poor reliability, changes in staff workload and changes associated with staff roles and responsibilities (24).

Poor usability of medical devices in particular is recognized as a major issue for patient health (76) and it is related to adverse events, patient injuries and readmission to hospital (77). Collaboration between physicians and experts in Human Factors and Ergonomics (HFE) is desirable for the design of medical devices to benefit not only patients but also HWs, formal and informal caregivers. However, at present, unfortunately there is no methodological uniformity among studies on the usability of medical devices because often the rapid and continuous evolution of medical devices exceeds the development goals covered by rules and standards (75).

To correct for poor reliability, a telecare system should be flexible enough to automatically detect fault conditions, notify the patient and the local intelligence unit of fault conditions, and be fail-safe (32).

Remote monitoring is an additional tool for implementing reliability in telemedicine. Evidence supported the benefits of remote monitoring in reducing hospitalization/re-hospitalization, improving patient drug compliance and improving health outcomes (66), and also during pre-hospitalization, i.e., preparatory procedures before hospital medical or surgical treatment, and after discharge, particularly for collecting Patient reported Outcome Measurements, which are now entering in the standardized evaluation of follow-up of the patient, to measure the improvement of quality of life linked to the medical treatment (78). The digital medicine should really improve the patient journey, decreasing economical and social costs of patient transfers, clearly evident and increasing in modern medicine, owing to centralization of some specialized treatments and technology.

Incident Reporting Systems (IRS) are a cornerstone of patient safety improvement (79). However, a recent review showed that patient safety initiatives in Health Information Technology (HIT) mainly concern software. Instead, more standardization and supervision is needed to ensure security throughout the lifecycle and initiatives should cover both software and hardware (80). Although the application of such patient safety initiatives in home care is a complex challenge (81, 82), reporting of adverse events should become mandatory as well as for medical devices.

In assessing the critical patient safety issues related to digitisation and automation, it is necessary to consider the enhancement of telemedicine through artificial intelligence (AI). AI will increasingly integrate with telemedicine (13) it will facilitate the use of telemedicine as a tool for the shift from hospital to home-based care (83).

At the same time, an integrated telemedicine-AI system will also incorporate the critical issues of AI: “black box” problem and unclear definition of liability for AI-related errors and damages.

The latest machine learning models are like “black boxes,” i.e., they have such a complex structure that users cannot understand how an AI system converts data into decisions-making (84). However, human-computer interaction forms an Integrated Cognitive System in which the human operator remains at the top of the system and can take over when a specific situation requires it (85, 86).

Uncontrolled and incorrect decision-making by an algorithm can cause serious and irreparable damage. This is a risk that no health care activity can afford. Certainly not telemedicine where human supervision in accordance with an Integrated Cognitive System may be limited as the HWs and the patient are not in the same location.

Another issue concerns the liability in the event that the operation or malfunction of an AI system causes harm to a human being.

A recent resolution of the European Parliament stated that a human being, and not a robot, should be responsible at the moment. The resolution specified that the greater the learning capacity or autonomy of a robot and the longer the duration of a robot's training, the greater should be the responsibility of its trainer. However, in determining actual liability for the damage caused, the skills resulting from the 'training' of a robot should not be confused with skills that depend strictly on its self-learning abilities. It was therefore proposed that the most sophisticated autonomous robots could be considered as electronic persons responsible for compensating any damage caused by them (87).

The European Economic and Social Committee (EESC) is opposed to the introduction of legal personality for robots or AI systems, as this would nullify the preventive function of correcting behavior once civil liability no longer falls on the manufacturer because it has been transferred to the robot or AI system (88).

For criminal law, the liability of artificial intelligence systems is still very much unexplored territory, but it is not without interest. It has been argued that intelligent autonomous agents with cognitive and machine-learning capabilities should not be considered as mere devices (89).

The increasing integration of AI in telemedicine requires absolute transparency between physicians and patients, between physicians and healthcare organizations and between healthcare organizations and the community because it is essential for quality, safety, accountability and informed decision-making (90).

Patients are increasingly willing to adopt telemedicine systems but their compliance with existing regulations needs to be implemented and responsibilities for all parties involved need to be better defined (91). In addition, a standardization or almost a homogenization of processes used in telemedicine should be defined a priori, through the classical and universally accepted scientific approach and validation, i.e., case-control and/or randomized studies and/or health technology assessment, to assure quality and efficacy of the web-based procedures. In fact, the procedures admitted and applied are widely different, although contained in a “digital area,” considering also different cognitive involvement of the patient and different technological equipment; the evidences of efficacy of telemedicine, are still few, despite the wide practical use of this approach, just for example in tele-rehabilitation after orthopedic surgery (92).

Finally, another structural factor must be added to these variables, namely that healthcare is not provided in a hospital but at the patient's home through the use of digital technologies. There are no standards concerning the physical environment in which home care services are provided, in stark contrast to the requirements for healthcare institutions (35).

However, this peculiar kind of health care requires a human-systems approach to understand the interactions between people, equipment/technology, tasks and environments (93). In this perspective, promoting specific improvements in the area of patient safety for telemedicine would require the home to be considered as a complex working system in which different human, technological and environmental factors interact to influence the health care process (24).

## Position Statement

The critical issues related to the use and diffusion of telemedicine call for a wide-ranging reflection on medical, legal, ethical and organizational principles in order to provide safe health and social care.

Clinical Risk Management aims to improve the safety of care by identifying and preventing circumstances that could expose a patient to the risk of an adverse event.

Intrinsic ethical implications are present throughout the clinical risk management process, i.e., in the assessment, management and communication of clinical risk. Indeed, from an ethical point of view, risks can be approached from different perspectives (utilitarian, contractual, subjective, sociobiological and personalistic) (74).

A literature review showed that the ethical aspects of specific telemedicine applications are a neglected area, with only a few empirical studies (94). However, there are many ethical issues related to telemedicine, many of which are addressed in this paper: the protection of patient autonomy and the right to express informed consent to the proposed treatment; the appropriateness of telemedicine in relation to the specific clinical case; the proper identification of the patient; the guarantee of equal access to treatment and quality care; the definition of professional duties and responsibilities; the preservation and integrity of confidential patient data; the dehumanization of healthcare (95). Ethical principles are experienced differently by telecare providers and patients: providers consider that telemedicine provides better care than patients; patients feel that telemedicine may place a greater share of costs and burdens on them, reducing equity (96).

The World Medical Association and the American Medical Association have endorsed the need to create an ethically-based system that safeguards the interest of patients and reduces the risks of non-compliance and compromised effectiveness (58, 59).

**Figure 2:**
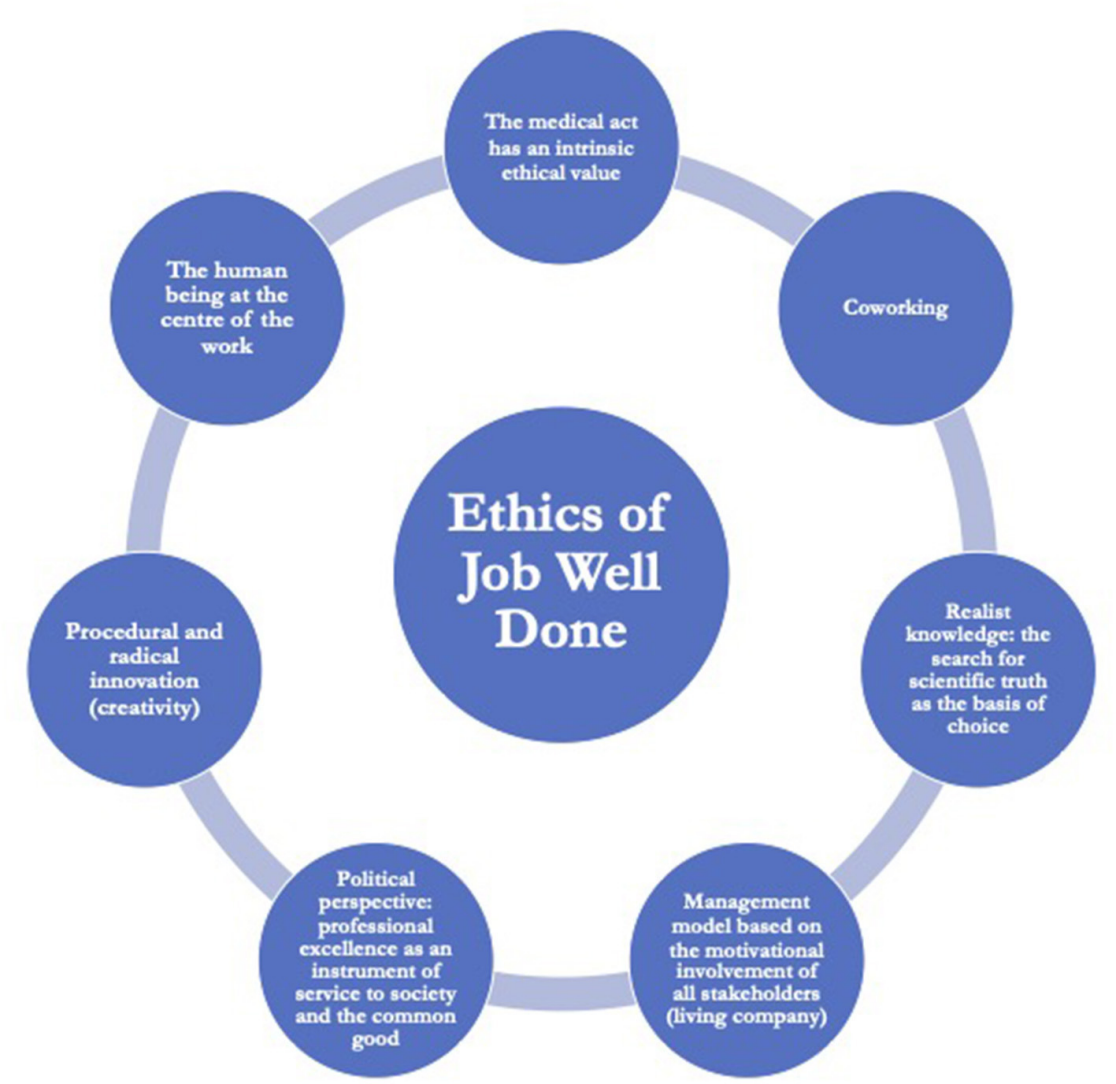
Ethics of Job Well Done framework.

For this reason, let us first define our perspective. The ethical framework we refer to is the Ethics of Job Well Done, which is part of ethical personalism (29). According to the Ethics of Job Well Done, health care in telemedicine should be characterized by: (a) an awareness that every medical act is a free and responsible Human Act with an intrinsic ethical value; (b) an interdisciplinary co-design in relation to complexity theory and systemic thinking; (c) a realistic knowledge that always starts from experience and leads to the search for scientific truth as the basis for one's choices; (d) a management model useful for the motivational involvement of all the components involved; (e) a recovery of the political dimension of Job Well Done, i.e., professional excellence as a means of serving society and the common good; (f) the capacity for radical procedural innovation; (g) putting people at the center of work, improving effectiveness and efficiency and ensuring sustainability (Figure 2).

**Figure 3:**
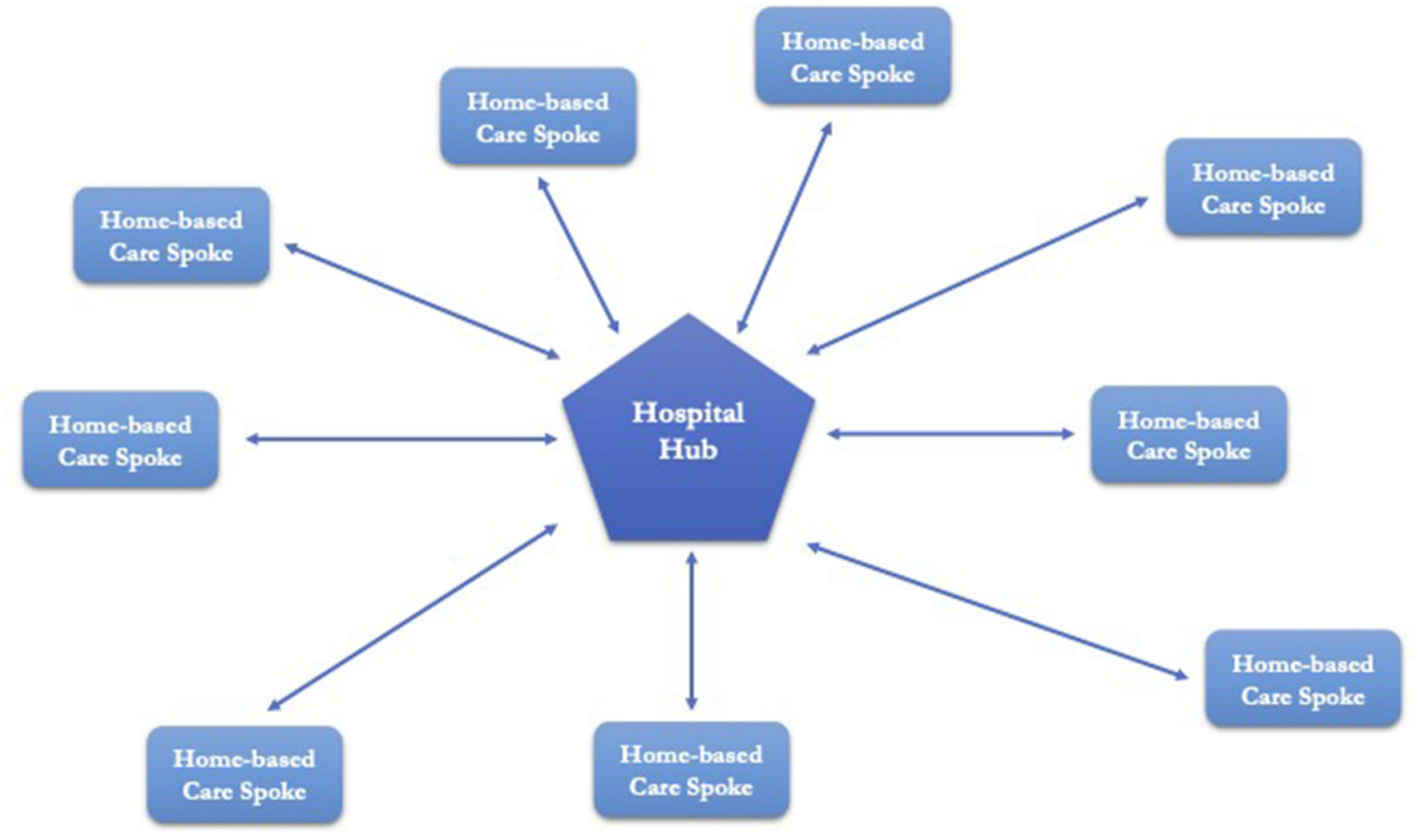
“Hub and Spoke” organizational model.

Based on the Ethics of Job Well Done, we propose the following lines of action:

1. The telemedicine service should be integrated in a “Hub and Spoke” organizational model, where the hospital is the Hub and the patient's home is the Spoke. This would make it possible to identify, assess and eliminate current and potential risks in the healthcare process carried out through telemedicine in order to ensure the quality and safety of care services (Figure 3).
2. As an integral part of healthcare (Hub and Spoke), HWs, formal and informal caregivers should have an incident reporting system in place to collect information about the occurrence of adverse events, near misses or sentinel events.
3. CRM should be based on a systems approach. Systemic Clinical Risk Management (SCRM) is a proactive CRM approach in which patient safety is the result of the acquisition and processing of multifactorial quantitative e-tech data and qualitative data such as the human-animal-environment interface, lifestyle behaviors, social factors, political and socio-economic conditions and globalization processes (97). The systemic approach to patient safety should be fostered by the increasing implementation and integration of AI systems and telemedicine.
4. HWs, formal and informal caregivers should receive specific training on critical telemedicine issues regarding risk management in telemedicine, communication processes and critical topics such as confidentiality, security, privacy, storage and integrity of sensitive data. The aim is to implement a culture of safety and to achieve an adequate and specific level of competence for each of the players involved in the healthcare procedure.
5. Patients should be selected carefully. It would be necessary to verify the correspondence between the patient's health needs and the possibility of delivering the service *via* telemedicine. The aim is to pursue appropriateness of provision (supporting, supplementing or replacing telemedicine) by also using tools for remote detection and monitoring of biological parameters and clinical surveillance.
6. Health care through telemedicine should only be started after an initial in-person medical consultation, except for specific needs determined by the physician and for situations that make the patient's presence in a health facility particularly difficult (mobility problems, impossibility of finding an accompanying person, etc.).
7. Each patient should have a medical *case manager* to refer to.
8. The patient should be able to check and communicate to the *case manager* the degree of satisfaction and should be able to communicate any need or desire for change.
9. Healthcare organizations should identify an appropriate catchment area, clinical pathways, structural and organizational arrangements, and rules regarding transfers from hospital/hub to home-based care/Spoke and vice versa in case of need.
10. Informed consent should fully explain the process of treatment through telemedicine, expected outcomes, non-treatment outcomes, complications, treatment alternatives, impact on family life, organizational, structural and instrumental needs, impact and risks to privacy. The language should be comprehensible, simple and clear, appropriate to the age, capacity and health, psychological, cultural and linguistic status of the patient. The aim is to provide the patient with as much information as possible so that the choice to be assisted by telemedicine is a free and informed one and not an obligation without an alternative. In this way, the principle of autonomy would not conflict with the principle of health protection.
11. The implementation in medical practice of telemedicine procedures should be defined by the evaluation and validation of efficacy obtained from specific studies performed following classical scientific schemes and also by metanalyses and health technology assessment.
12. Patients' organizations, especially disabled and vulnerable ones, should participate in decision-making processes and they should help healthcare organizations to monitor the long-term effects of telemedicine.
13. Information and Communication Technologies (ICT) must guarantee authentication, safety, security, integrity, confidentiality and availability of data.

These lines of action can help stakeholders involved in safety promotion to manage telemedicine risk-related, i.e., patients and their relatives, formal and informal caregivers, patients' organizations, policy-makers, healthcare governance, healthcare workers (HWs), providers and insurance providers.

## Conclusion

During the pandemic, telemedicine was the tool through which many Health Systems ensured health care for Covid-19 patients with mild symptoms and for patients who needed diagnosis and treatment but had to respect physical distancing (8–12).

Because of its recognized effectiveness, many countries have planned economic and regulatory interventions (15–18) to enhance telemedicine even after the pandemic with the aim of moving healthcare from the hospital to the territory, strengthening healthcare and improving standards of patient care.

Nevertheless, telemedicine is not a new healthcare tool. A 1997 paper paradigmatically titled “Telemedicine: new technology = new questions = new exposures” (98).

Simply the Covid-19 pandemic has emphasized its usefulness. Telemedicine is experiencing a new springtime. The hope is that telemedicine does not fall into oblivion or, worse still, become a boomerang for the health and safety of patients, the safety of all professionals and others, the expectations of family members, and also for the budgets of health systems worldwide.

For this not to happen, we need to put into practice some “alerts” already sounded in the pre-Covid-19 era.

In 2004, the Commission of the European Communities argued that e-Health should be supported by a wide diffusion of best practices including quality impact and accountability assessments in telemedicine services and accreditation procedures (99). Eight years later, for professionals (health and scientific communities) the focus will be on developing evidence-based clinical practice guidelines for telemedicine services with particular emphasis on nursing and social care workers (100).

In 2005, the WHO supported the need to diffuse experiences and best practices regarding telemedicine, to promote standards through the diffusion of guidelines and to train HWs to strengthen the quality and safety of healthcare (26).

In 2018, the WMA's position steatment encouraged the development of ethical standards, practice guidelines, national legislation and international agreements on topics related to the practice of telemedicine, protecting the patient-physician relationship, confidentiality and quality of medical care (58).

The American Medical Association (AMA) has supported the continuous improvement of telehealth/telemedicine technologies, and the development and implementation of clinical and technical standards to ensure safety and quality of care (59).

Telemedicine is a major challenge for all healthcare organizations wishing to offer telemedicine programmes to patients. Telemedicine programmes are positioned within larger health organizations and do not operate in a vacuum. In turn, each organization operates within a wider environment, which is often limited by fiscal, geographical and personnel factors. All these factors could influence the introduction of telemedicine (101).

This enormous task requires new knowledge which, if not recognized, could see telemedicine projects continue to founder (102).

With this position paper we have accepted the challenge and wanted to make our own contribution. We are convinced that the sustainable development of telemedicine can only be achieved by increasing citizens' confidence in telemedicine. To do so, healthcare organizations will have to offer quality, technologically advanced and safe healthcare for patients, professional and non-professional stakeholders involved in the process of care.

## Data Availability Statement

The original contributions presented in the study are included in the article/supplementary materials, further inquiries can be directed to the corresponding author.

## Author Contributions

FD, LP, and ER: writing. VF: supervision. GB, AO, GC, and RA: revision. PF and TP: writing revision. GT: data curation. MP: visualization. AD and VT: conceptualization. All authors contributed to the article and approved the submitted version.

## Conflict of Interest

The authors declare that the research was conducted in the absence of any commercial or financial relationships that could be construed as a potential conflict of interest.

## Publisher's Note

All claims expressed in this article are solely those of the authors and do not necessarily represent those of their affiliated organizations, or those of the publisher, the editors and the reviewers. Any product that may be evaluated in this article, or claim that may be made by its manufacturer, is not guaranteed or endorsed by the publisher.

## References

[1] World Health Organization. A Health Telematics Policy in Support of WHO's Health-For-All Strategy for Global Health Development: Report of the WHO Group Consultation on Health Telematics. (1998). Available online at: https://apps.who.int/iris/handle/10665/63857 (accessed March 15, 2022).

[2] Ferorelli D Nardelli L Spagnolo L Corradi S Silvestre M Misceo F. Medical legal aspects of telemedicine in Italy: application fields, professional liability and focus on care services during the COVID-19 health emergency. J Prim Care Community Health. (2020) 11:2150132720985055. 10.1177/2150132720985055 33372570 PMC7780315

[3] Bhaskar S Bradley S Chattu VK Adisesh A Nurtazina A Kyrykbayeva S. Telemedicine across the globe-position paper from the COVID-19 pandemic health system resilience PROGRAM (REPROGRAM) international consortium (part 1). Front Public Health. (2020) 8:556720. 10.3389/fpubh.2020.556720 33178656 PMC7596287

[4] Commission of the European Communities. Communication From the Commission to the European Parliament, the Council, the European Economic and Social Committee and the Committee of the Regions. Telemedicine for the Benefit of Patients, Healthcare Systems and Society. (2008). Available online at: https://ec.europa.eu/transparency/documents-register/detail?ref=COM(2008)689&lang=it (accessed March 15, 2022).

[5] European Commission,. Communication From the Commission to the European Parliament, the Council, the European Economic Social Committee the Committee of the Regions. A Digital Agenda for Europe. (2010). Available online at: https://eufordigital.eu/wp-content/uploads/2019/10/COMMUNICATION-FROM-THE-COMMISSION-TO-THE-EUROP EAN-PARLIAMENT.pdf (accessed March 15, 2022).

[6] European Commission,. Communication From the Commission to the European Parliament, the Council, the European Economic Social Committee the Committee of the Regions on Enabling the Digital Transformation of Health Care in the Digital Single Market; Empowering Citizens Building a Healthier Society. (2018). Available online at: https://eur-lex.europa.eu/legal-content/EN/TXT/PDF/?uri=CELEX:52018DC0233&from=EN (accessed March 15, 2022).

[7] Hyder MA Razzak J. Telemedicine in the United States: an introduction for students and residents. J Med Internet Res. (2020) 22:e20839. 10.2196/20839 33215999 PMC7690251

[8] Monaghesh E Hajizadeh A. The role of telehealth during COVID-19 outbreak: a systematic review based on current evidence. BMC Public Health. (2020) 20:1193. 10.1186/s12889-020-09301-4 32738884 PMC7395209

[9] Bhaskar S Bradley S Chattu VK Adisesh A Nurtazina A Kyrykbayeva S. Telemedicine as the new outpatient clinic gone digital: position paper from the pandemic health system REsilience PROGRAM (REPROGRAM) international consortium (part 2). Front Public Health. (2020) 8:410. 10.3389/fpubh.2020.00410 33014958 PMC7505101

[10] Bhaskar S Nurtazina A Mittoo S Banach M Weissert R. Editorial: telemedicine during and beyond COVID-19. Front Public Health. (2021) 9:662617. 10.3389/fpubh.2021.662617 33796502 PMC8007781

[11] World Health Organization. Regional Office for Europe, Strengthening the Health Systems Response to COVID-19: Policy Brief: Recommendations for the WHO European Region. (2020). Available online at: https://apps.who.int/iris/handle/10665/333072 (accessed March 15, 2022).

[12] The Organisation for Economic Co-operation Development (OECD). Beyond Containment: Health Systems Responses to COVID-19 in the OECD. (2020). Available online at: https://www.oecd.org/coronavirus/policy-responses/beyond-containment-health-systems-respon ses-to-covid-19-in-the-oecd-6ab740c0/#blocknotes-d7e786 (accessed March 15, 2022).

[13] Bhaskar S Bradley S Sakhamuri S Moguilner S Chattu VK Pandya S. Designing futuristic telemedicine using artificial intelligence and robotics in the COVID-19 era. Front Public Health. (2020) 8:556789. 10.3389/fpubh.2020.556789 33224912 PMC7667043

[14] European Union,. Regulation (EU) 2021/522 of the European Parliament of the Council of 24 March 2021 Establishing a Programme for the Union's Action in the Field of Health (‘EU4Health Programme') for the Period 2021-2027, Repealing Regulation (EU) No 282/2014. (2021). Available online at: https://eur-lex.europa.eu/legal-content/EN/TXT/?uri=uriserv:OJ.L_.2021.107.01.0001.01.ENG (accessed March 15, 2022).

[15] Governo italiano,. Piano Nazionale di Ripresa e Resilienza. (2021). Available online at: https://www.governo.it/sites/governo.it/files/PNRR.pdf (accessed March 15, 2022).

[16] Gouvernement français,. Plan National de Relance et de Résilience. (2021). Available online at: https://www.economie.gouv.fr/plan-national-de-relance-et-de-resilience-pnrr (accessed March 15, 2022).

[17] Bunderministerium der Finanzen,. Deutscher Aufbau-und Resilienzplan. (2021). Available online at: https://www.bundesfinanzministerium.de /Content/DE/Standardartikel/ Themen/ Europa/ DARP/deutscher-aufbau-und-resilienzplan.html (accessed March 15, 2022).

[18] The United States Congress. Telehealth Extension and Evaluation Act. (2022). Available online at: https://www.cortezmasto.senate.gov/imo/media/doc/GOE22074.pdf (accessed March 15, 2022).

[19] Aly DM Erickson LA Hancock H Apperson JW Gaddis M Shirali G. Ability of video telemetry to predict unplanned hospital admissions for single ventricle infants. J Am Heart Assoc. (2021) 10:e020851. 10.1161/JAHA.121.020851 34365801 PMC8475020

[20] Insulander P Carnlöf C Schenck-Gustafsson K Jensen-Urstad M. Device profile of the Coala Heart Monitor for remote monitoring of the heart rhythm: overview of its efficacy. Expert Rev Med Devices. (2020) 17:159–65. 10.1080/17434440.2020.1732814 32101067

[21] The Joint Commission releases improving America's hospitals: the Joint Commission's annual report on quality and safety 2007. Jnt Comm Perspect. (2007) 27:1–3. 18257444

[22] World Health Organization. Patient Safety Curriculum Guide: Multi-Professional Edition. (2011). Available online at: https://www.who.int/patientsafety/education/curriculum/who_mc_topic-6.pdf (accessed March 15, 2022).

[23] Solimini R Busardò FP Gibelli F Sirignano A Ricci G. Ethical and legal challenges of telemedicine in the era of the COVID-19 pandemic. Medicina. (2021) 30:1314. 10.3390/medicina57121314 34946259 PMC8705012

[24] Guise V Anderson J Wiig S. Patient safety risks associated with telecare: a systematic review and narrative synthesis of the literature. BMC Health Serv Res. (2014) 14:588. 10.1186/s12913-014-0588-z 25421823 PMC4254014

[25] Darkins A. Patient safety considerations in developing large telehealth networks. Clin Risk. (2012) 18:90–4. 10.1258/cr.2012.012006

[26] World Health Organization. Resolutions and Decisions - WHA58.28 on eHealth. (2005). Available online at: https://www.who.int/healthacademy/media/WHA58-28-en.pdf (accessed March 15, 2022).

[27] NEJM Catalyst,. What Is Risk Management in Healthcare? (2018). Available online at: https://catalyst.nejm.org/doi/full/10.1056/CAT.18.0197 (accessed May 10, 2022).

[28] American Society for Health Care Risk Management. Enterprise Risk Management. (2021). Available online at: https://www.ashrm.org/system/files?file=media/file/2019/06/ERM-Tool_final.pdf (accessed May 10, 2022).

[29] Quintiliani L Sisto A Vicinanza F Curcio G Tambone V. Resilience and psychological impact on Italian university students during COVID-19 pandemic. Distance learning and health. Psychol Health Med. (2022) 27:69–80. 10.1080/13548506.2021.1891266 33602027

[30] Card AJ. What is ethically informed risk management? AMA J Ethics. (2020) 22:E965–75. 10.1001/amajethics.2020.965 33274710

[31] American Society for Health Care Risk Management. Health Care Risk Management Code of Professional Responsibility. (2013). Available online at: https://www.ashrm.org/sites/default/files/ashrm/Code_of_Conduct_2013.pdf (accessed May 10, 2022).

[32] Williams G Doughty K Bradley DA. Safety and risk issues in using telecare. J Telemed Telecare. (2000) 6:249–62. 10.1258/1357633001935833 11070585

[33] Institute Institute of Medicine (US) Committee on Quality of Health Care in America Kohn LT Corrigan JM Donaldson MS editors. To Err is Human: Building a Safer Health System. Washington, DC: National Academies Press (2000). 25077248

[34] Carayon P Wetterneck TB Rivera-Rodriguez AJ Hundt AS Hoonakker P Holden R. Human factors systems approach to healthcare quality and patient safety. Appl Ergon. (2014) 45:14–25. 10.1016/j.apergo.2013.04.023 23845724 PMC3795965

[35] Lang A Edwards N Fleiszer A. Safety in home care: a broadened perspective of patient safety. Int J Qual Health Care. (2008) 20:130–5. 10.1093/intqhc/mzm068 18158294

[36] Sackett DL Rosenberg WM Gray JA Haynes RB Richardson WS. Evidence based medicine: what it is and what it isn't. BMJ. (1996) 13:71–2. 10.1136/bmj.312.7023.71 8555924 PMC2349778

[37] Karsh BT Holden RJ Alper SJ Or CK. A human factors engineering paradigm for patient safety: designing to support the performance of the healthcare professional. Qual Saf Health Care. (2006) 15:i59–65. 10.1136/qshc.2005.015974 17142611 PMC2464866

[38] Romagnoli KM Handler SM Hochheiser H. Home care: more than just a visiting nurse. BMJ Qual Saf. (2013) 22:972–4. 10.1136/bmjqs-2013-002339 23940375 PMC4120108

[39] Johnson KG. Adverse events among Winnipeg home care clients. Healthc Q. (2006) 9:127–34. 10.12927/hcq.2013.18377 17087182

[40] Blais R Sears NA Doran D Baker GR Macdonald M Mitchell L. Assessing adverse events among home care clients in three Canadian provinces using chart review. BMJ Qual Saf. (2013) 22:989–97. 10.1136/bmjqs-2013-002039 23828878 PMC3888609

[41] Donabedian A. Explorations in Quality Assessment and Monitoring: The Definition of Quality and Approaches to its Assessment. Ann Arbor: Michigan Health Administration Press (1980).

[42] Carayon P Schoofs Hundt A Karsh BT Gurses AP Alvarado CJ Smith M. Work system design for patient safety: the SEIPS model. Qual Saf Health Care. (2006) 15:i50–8. 10.1136/qshc.2005.015842 17142610 PMC2464868

[43] Stange KC Nutting PA Miller WL Jaén CR Crabtree BF Flocke SA. Defining and measuring the patient-centered medical home. J Gen Intern Med. (2010) 25:601–12. 10.1007/s11606-010-1291-3 20467909 PMC2869425

[44] Wetterneck TB Lapin JA Karsh BT Beasley JW. Human factors and ergonomics in primary care. In: Carayon P, editor. Handbook of Human Factors and Ergonomics in Health Care and Patient Safety. Boca Raton, FL: Taylor and& Francis Group (2012) p. 763–74.

[45] Botrugno C. Towards an ethics for telehealth. Nurs Ethics. (2019) 26:357–67. 10.1177/0969733017705004 28502219

[46] Marcinowicz L Konstantynowicz J Godlewski C. Patients' perceptions of GP non-verbal communication: a qualitative study. Br J Gen Pract. (2010) 60:83–7. 10.3399/bjgp10X483111 20132701 PMC2814260

[47] Pinto RZ Ferreira ML Oliveira VC Franco MR Adams R Maher CG. Patient-centred communication is associated with positive therapeutic alliance: a systematic review. J Physiother. (2012) 58:77–87. 10.1016/S1836-9553(12)70087-5 22613237

[48] Leonard M Graham S Bonacum D. The human factor: the critical importance of effective teamwork and communication in providing safe care. Qual Saf Health Care. (2004) 13:i85–90. 10.1136/qshc.2004.010033 15465961 PMC1765783

[49] Ciccozzi M Menga R Ricci G Vitali MA Angeletti S Sirignano A. Critical review of sham surgery clinical trials: Confounding factors analysis. Ann Med Surg (Lond). (2016) 12:21–6. 10.1016/j.amsu.2016.10.007 27872745 PMC5109256

[50] Nittari G Khuman R Baldoni S Pallotta G Battineni G Sirignano A. Telemedicine practice: review of the current ethical and legal challenges. Telemed J E Health. (2020) 26:1427–37. 10.1089/tmj.2019.0158 32049608 PMC7757597

[51] Kaplan B. Revisiting health information technology ethical, legal, and social issues and evaluation: telehealth/telemedicine and COVID-19. Int J Med Inform. (2020) 143:104239. 10.1016/j.ijmedinf.2020.104239 33152653 PMC7831568

[52] O'Daniel M Rosenstein AH. Professional Communication and Team Collaboration. In: Hughes RG, editor. Patient Safety and Quality: An Evidence-Based Handbook for Nurses. Rockville, MD: Agency for Healthcare Research and Quality (2008). 21328739

[53] Agha Z Roter DL Schapira RM. An evaluation of patient-physician communication style during telemedicine consultations. J Med Internet Res. (2009) 11:e36. 10.2196/jmir.1193 19793720 PMC2802255

[54] Street R Wheeler J McCaughan W. Specialist-primary care provider-patient communication in telemedical consultations. Telemed J. (2000) 6:45–54. 10.1089/107830200311842

[55] Miller EA. Telemedicine and doctor-patient communication: an analytical survey of the literature. J Telemed Telecare. (2001) 7:1–17. 10.1258/1357633011936075 11265933

[56] Morony S Weir K Duncan G Biggs J Nutbeam D Mccaffery KJ. Enhancing communication skills for telehealth: development and implementation of a Teach-Back intervention for a national maternal and child health helpline in Australia. BMC Health Serv Res. (2018) 7:162. 10.1186/s12913-018-2956-6 29514642 PMC5842621

[57] Le cure primarie PD. In: Tartaglia R, Vannucci A, editors. Prevenire gli eventi avversi in medicina, Milano: Springer-Verlag Italia (2013).p. 241–54.

[58] World Medical Association. WMA Statement on The Ethics of Telemedicine. (2018). Available online at: https://www.wma.net/policies-post/wma-statement-on-the-ethics-of-telemedicine/ (accessed March 15, 2022).

[59] American Medical Association. Ethical Practice in Telemedicine. Available online at: https://www-ama--assn-org.translate.goog/delivering-care/ethics/ethical-practice-telemedicine? _x_tr_sl =en&_x_tr_tl=it&_x_tr_hl=it&_x_tr_pto=wapp (accessed March 15, 2022).

[60] European Commission,. Task Shifting Health System Design Report of the Expert Panel on Effective Ways of Investing in Health (EXPH). (2019). Available online at: https://ec.europa.eu/health/system/files/2019-11/023_taskshifting_en_0.pdf (accessed March 15, 2022).

[61] Bittner-Fagan H Davis J Savoy M. Improving patient safety: improving communication. FP Essent. (2017) 463:27–33. 29210557

[62] De Micco F Martino F Campobasso CP. Ethical issues in age assessment by the third molar development. Aust J Forensic Sci. (2020) 54:88–99. 10.1080/00450618.2020.1789220

[63] The Joint Commission International. Informed Consent: More Than Getting a Signature. (2016). Available online at: https://www.jointcommission.org/-/media/tjc/documents/newsletters/quick_safety_issue_twenty-one_february_2016pdf.pdf (accessed March 15, 2022).

[64] MedPro Group,. Risk Management Strategies for Informed Consent. (2020). Available online at: https://www.medpro.com/documents/10502/2837997/Guideline_Risk+Management+Strategies+for+Informed+Consent.pdf (accessed March 15, 2022).

[65] Green BE. Proactively Approaching Telehealth Informed Consent. (2013). Available online at: https://www.lexology.com /library/detail.aspx?g =a90e1cd6-8090-481b-90c0-7b19407a70a0 (accessed March 15, 2022).

[66] Gough F Budhrani S Cohn E Dappen A Leenknecht C Lewis B. ATA practice guidelines for live, on-demand primary and urgent care. Telemed J E Health. (2015) 21:233–41. 10.1089/tmj.2015.0008 25658882

[67] Simon CM Schartz HA Rosenthal GE Eisenstein EL Klein DW. Perspectives on electronic informed consent from patients underrepresented in research in the United States: a focus group study. J Empir Res Hum Res Ethics. (2018) 13:338–48. 10.1177/1556264618773883 29790410

[68] Grauberger J Kerezoudis P Choudhry AJ Alvi MA Nassr A Currier B. Allegations of failure to obtain informed consent in spinal surgery medical malpractice claims. JAMA Surg. (2017) 152:e170544. 10.1001/jamasurg.2017.0544 28445561 PMC5831424

[69] Raval T,. Fighting Telemedicine Fraud: Why Robust Verification is Needed. (2020). Available online at: https://www.forbes.com/sites/ forbestechcouncil/2020/08/25/fighting-telemedicine-fraud-why-robust-verification-is-needed/?sh=4422b8ed1f6f (accessed March 15, 2022).

[70] Terry M. Medical identity theft and telemedicine security. Telemed J E Health. (2009) 15:928–32. 10.1089/tmj.2009.9932 19908998

[71] Garg V Brewer J. Telemedicine security: a systematic review. J Diabetes Sci Technol. (2011) 5:768–77. 10.1177/193229681100500331 21722592 PMC3192643

[72] Hall JL McGraw D. For telehealth to succeed, privacy and security risks must be identified and addressed. Health Aff. (2014) 33:216–21. 10.1377/hlthaff.2013.0997 24493763

[73] Travaini G Caruso P Merzagora I. Crime in Italy at the time of pandemic. Acta Biomedica. (2020) 91:199. 10.23750/abm.v91i2.9596 32420945 PMC7569631

[74] Ministero della Salute,. Sicurezza dei pazienti e gestione del rischio clinico: Manuale per la formazione degli operatori sanitari. (2007). Available online at: https://www.salute.gov.it/imgs/C_17_pubblicazioni_640_allegato.pdf (accessed March 15, 2022).

[75] Ranzani F Parlangeli O. Digital technology and usability and ergonomics of medical devices. In: Donaldson L, Ricciardi W, Sheridan S, Tartaglia R, editors. Textbook of Patient Safety and Clinical Risk Management. Cham (2021). p. 455–64. 36315768

[76] U.S. Food and Drug Administration. Draft Guidance for Industry and Food and Drug Administration Staff – Applying Human Factors and Usability Engineering to Optimize Medical Device Design. (2011). Available online at: http://www.fda.gov/MedicalDevices/Device RegulationandGuidance/GuidanceDocuments/ucm 259748.htm (accessed March 15, 2022).

[77] Reed TL Kaufman-Rivi D. FDA adverse event problem codes: standardizing the classification of device and patient problems associated with medical device use. Biomed Instrum Technol. (2010) 44:248–56. 10.2345/0899-8205-44.3.248 20715359

[78] Cabitza F Dui LG. Collecting patient reported outcomes in the wild: opportunities and challenges. Stud Health Technol Inform. (2018) 247:36–40. 29677918

[79] Pham JC Gianci S Battles J Beard P Clarke JR Coates H. Establishing a global learning community for incident-reporting systems. Qual Saf Health Care. (2010) 19:446–51. 10.1136/qshc.2009.037739 20977995

[80] Magrabi F Aarts J Nohr C Baker M Harrison S Pelayo S. A comparative review of patient safety initiatives for national health information technology. Int J Med Inform. (2013) 82:139–48. 10.1016/j.ijmedinf.2012.11.014 23266061

[81] Goodman KW Berner ES Dente MA Kaplan B Koppel R Rucker D. Challenges in ethics, safety, best practices, and oversight regarding HIT vendors, their customers, and patients: a report of an AMIA special task force. J Am Med Inform Assoc. (2011) 18:77–81. 10.1136/jamia.2010.008946 21075789 PMC3005880

[82] Fu M Weick-Brady M Tanno E. Medical devices in the home: a unique challenge for the FDA. Work. (2012) 41:361–5. 10.3233/WOR-2012-1305 22398505

[83] World Health Organization. Ethics and Governance of Artificial Intelligence for Health: WHO guidance. (2021). Available online at: https://www.who.int/publications/i/item/9789240029200 (accessed March 15, 2022).

[84] Habli I Lawton T Porter Z. Artificial intelligence in health care: accountability and safety. Bull World Health Organ. (2020) 98:251–6. 10.2471/BLT.19.237487 32284648 PMC7133468

[85] Hollnagel E. Cognitive Reliability and Error Analysis Method (CREAM). Amsterdam, NL: Elsevier (1998).

[86] Sheridan T. Human supervisory control of robot systems. In: Proceedings IEEE International Conference on Robotics and Automation. San Francisco, CA: IEEE (1986). p. 808–12.

[87] European Parliament,. Civil Law Rules on Robotics. (2017). Available online at: https://www.europarl.europa.eu/doceo/document/TA-8-2017-0051_EN.html (accessed March 15, 2022).

[88] European Economic Social Committee. Opinion of the European Economic and Social Committee on “Artificial Intelligence-The Consequences of Artificial Intelligence on the (Digital) Single Market, Production, Consumption, Employment and Society”. (2017). Available online at: https://eur-lex.europa.eu/legal-content/EN/TXT/PDF/?uri=CELEX:52016IE5369&from=CS (accessed March 15, 2022).

[89] Hallevy G. The criminal liability of artificial intelligence entities - from science fiction to legal social control. Akron Law J. (2010) 4:171–201. 10.2139/ssrn.1564096

[90] Kaplan GS,. Building a Culture of Transparency in Health Care. (2018). Available online at: https://hbr.Org/2018/11/building-a-culture-of-transparency-in-health-care (accessed March 15, 2022).

[91] Parimbelli E Bottalico B Losiouk E Tomasi M Santosuosso A Lanzola G. Trusting telemedicine: a discussion on risks, safety, legal implications and liability of involved stakeholders. Int J Med Inform. (2018) 112:90–8. 10.1016/j.ijmedinf.2018.01.012 29500027

[92] Wang Q Lee RL Hunter S Chan SW. The effectiveness of internet-based telerehabilitation among patients after total joint arthroplasty: a systematic review and meta-analysis of randomised controlled trials. J Telemed Telecare. (2021). 10.1177/1357633X20980291. [Epub ahead of print]. 33459120

[93] Beer JM McBride SE Mitzner TL Rogers WA. Understanding challenges in the front lines of home health care: a human-systems approach. Appl Ergon. (2014) 45:1687–99. 10.1016/j.apergo.2014.05.019 24958610 PMC4180111

[94] MethoTelemed, project,. The MAST Manual. Available online at: https://joinup.ec.europa.eu/sites/default/files/document/2014-12/The%20Model%20for%20Assessment%20of%20Telemedicine%20%28MAST%29%20Manual.pdf (accessed May 10, 2022).

[95] Cordeiro JV. Digital technologies and data science as health enablers: an outline of appealing promises and compelling ethical, legal, and social challenges. Front Med. (2021) 8:647897. 10.3389/fmed.2021.647897 34307394 PMC8295525

[96] Keenan AJ Tsourtos G Tieman J. Promise and peril-defining ethical telehealth practice from the clinician and patient perspective: a qualitative study. Digit Health. (2022) 8:1–17. 10.1177/20552076211070394 35024158 PMC8744182

[97] De Micco F De Benedictis A Fineschi V Frati P Ciccozzi M Pecchia L. From syndemic lesson after COVID-19 pandemic to a “systemic clinical risk management” proposal in the perspective of the ethics of job well done. Int J Environ Res Public Health. (2021) 19:15. 10.3390/ijerph19010015 35010289 PMC8750949

[98] Abke A Mouse-Young D. Telemedicine: new technology = new questions = new exposures. J Healthc Risk Manag. (1997) 17:3–6. 10.1002/jhrm.5600170402 10169963

[99] Commission of the European Communities. Communication From the Commission to the European Parliament, the Council, the European Economic and Social Committee and the Committee of the Regions e-Health - Making Healthcare Better for European Citizens: An Action Plan for a European e-Health Area. (2004). Available online at: https://eur-lex.europa.eu/LexUriServ/LexUriServ.do?uri=COM:2004:0356:FIN:EN:PDF (accessed March 15, 2022).

[100] Commission of the European Communities. Communication from the Commission to the European Parliament, the Council, the European Economic and Social Committee and the Committee of the Regions. eHealth Action Plan 2012-2020 - Innovative Healthcare for the 21st Century. (2012). Available online at: https://ec.europa.eu/transparency/documents-register/detail?ref=COM(2012)736&lang=it (accessed March 15, 2022).

[101] Whitten P Adams I. Success and failure: a case study of two rural telemedicine projects. J Telemed Telecare. (2003) 9:125–9. 10.1258/135763303767149906 12877772

[102] Mort M May CR Williams T. Remote doctors and absent patients: acting at a distance in telemedicine? Sci Technol Human Values. (2003) 28:274–95. 10.1177/0162243902250907

